# The Brief Case: Multisystem extrapulmonary manifestations associated with *Chlamydia psittaci* infection—a diagnostic exploration of an elderly male

**DOI:** 10.1128/jcm.00320-26

**Published:** 2026-08-12

**Authors:** Zhengzhe Sun, Shan Jin, Xiang Fang

**Affiliations:** 1 The First Affiliated Hospital of Anhui University of Chinese Medicine 658190, Hefei, Anhui, China; 2 Key Laboratory of Xin'an Medicine, Ministry of Education, Anhui University of Chinese Medicine 668186, Hefei, Anhui, China; Mayo Clinic Minnesota, Rochester, Minnesota, USA

**Keywords:** *Chlamydia* infection, tNGS, the unilateral high intraocular pressure, psittacosis pneumonia

## CASE

A 54-year-old male presented with persistent distending pain in the right frontal-parietal region, accompanied by right ocular distending pain and blurred vision. Two days later, the patient developed a low-grade fever with a self-measured temperature of 38.5°C, accompanied by an occasional mild cough. His medical history was notable for a cerebral hemorrhage 1 year earlier, leaving residual impaired mobility on the right side.

**Fig 1:**
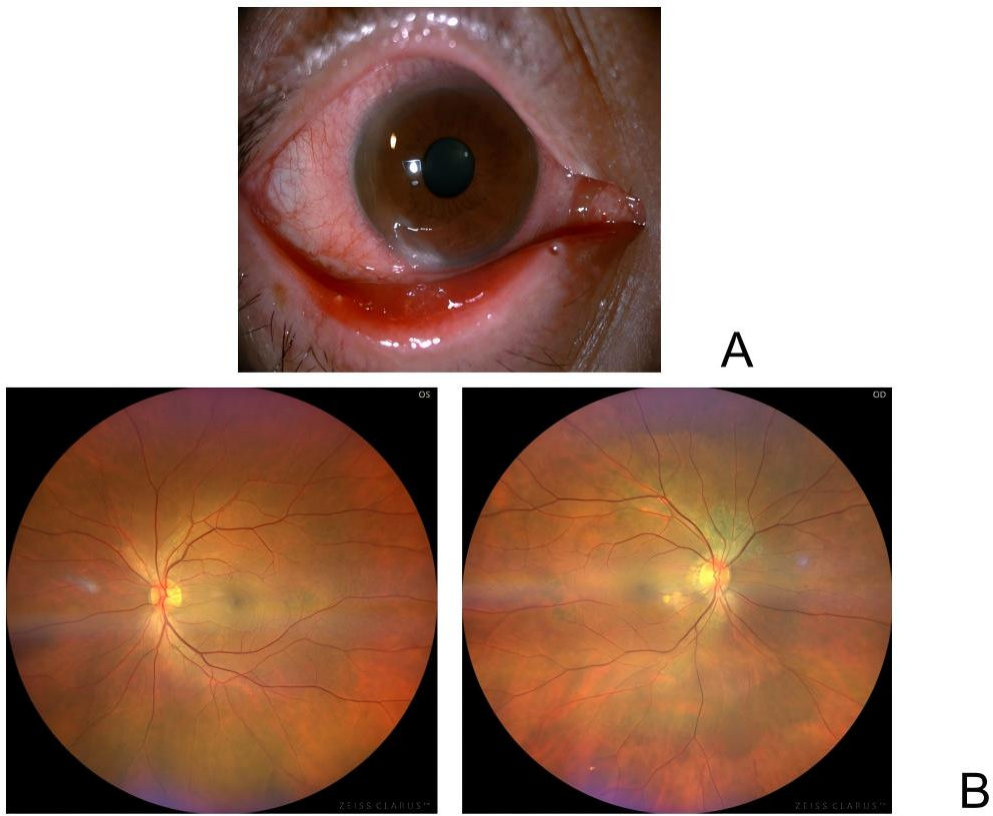
(**A**) The patient has conjunctival congestion in the right eye. (**B**) Patient’s binocular fundus image. The right optic disc boundary is clear, with a pale color and a disorder of retinal pigment. The optic disc boundary of the left eye is clear, with a pale color, and the retina is flat.

On admission, neurological examination was largely unremarkable, aside from right-sided spasticity secondary to his previous stroke. Ophthalmic examination revealed marked conjunctival congestion in the right eye (Fig. 1A). Visual acuity was 0.8 in the right eye (VOD) and 1.0 in the left (VOS). Intraocular pressure (IOP) was significantly elevated in the right eye at 30 mmHg, compared to 21 mmHg in the left. Fundus photography showed thinning of the right retinal layer (Fig. 1B). The patient was diagnosed with “ocular hypertension” and prescribed Cardiotenol eye drops, Brinzolamide eye drops, and mannitol. After treatment, the patient’s blurred vision and eye swelling improved slightly, but the right-sided headache persisted.

Laboratory investigations revealed a lymphocyte count of 0.55 × 10⁹/L (normal range: 1.1–3.2 × 10⁹/L), a lymphocyte percentage of 8.2% (normal range: 20%–50%), and serum sodium of 134.4 mmol/L (normal range: 137–145 mmol/L). Inflammatory markers were notably elevated: C-reactive protein, 84.63 mg/L (normal range: <10 mg/L); interleukin-6, 40.4 pg/mL (normal range: ≤7 pg/mL); and serum amyloid A, >300 mg/L (normal range: <10 mg/L). Procalcitonin was mildly elevated at 0.09 ng/mL (normal range: <0.05 ng/mL). A lumbar puncture yielded cerebrospinal fluid (CSF) with normal opening pressure, cell count, protein, and glucose levels. CSF microbiological cultures, stains, and polymerase chain reaction (PCR) for common neurotropic viruses (HSV, CMV, and EBV), as well as autoimmune encephalitis panels, were all negative. The patient’s CSF examination results were all normal, ruling out central nervous system (CNS) infection and autoimmune encephalitis.

**Fig 2:**
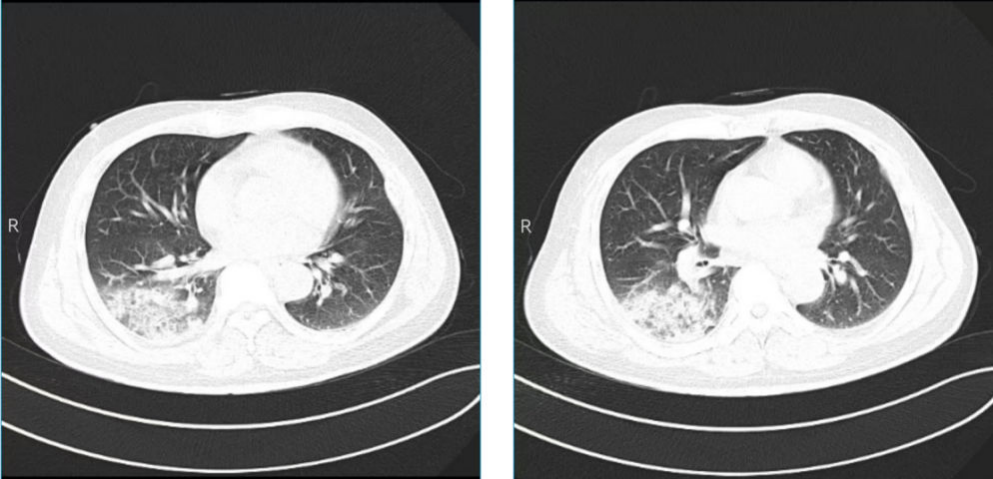
Representative chest CT image of the patient. Multiple patchy ground-glass opacities were visible in the lower lobe of the right lung, along with patchy consolidation, partial air-space consolidation, thickened interlobular septa, and air bronchogram. A small amount of pleural effusion was present. A small nodular shadow measuring approximately 4 mm in the longest diameter was visible in the lower lobe of the left lung.

Chest CT demonstrated multiple patchy ground-glass opacities and consolidation in the right lower lobe, thickened interlobular septa, an air-bronchogram, and bilateral pleural thickening with a small effusion (Fig. 2). Based on the patient’s clinical presentation, hematological inflammatory markers, and pulmonary imaging findings, the headaches and blurred vision were considered clinical symptoms caused by other systemic diseases.

Initial standard-of-care microbiological testing of the bronchoalveolar lavage fluid (BALF), including Gram stain, acid-fast stain, fungal smear, and conventional bacterial/fungal/mycobacterial cultures, yielded negative results. To definitively identify the etiology, targeted next-generation sequencing (tNGS) was performed on BALF collected 6 days after onset. The tNGS results identified *Chlamydia psittaci* with a specific sequence count of 30,062, normalized reads of 104,022 reads per million (RPM), and a high pathogen load of 6.39 × 10^4^ copies/mL. Upon re-evaluating the patient’s history, he disclosed recent close contact with live poultry raised near his residence.

He was ultimately diagnosed with psittacosis pneumonia with secondary extrapulmonary manifestations. Treatment was transitioned to intravenous omadacycline (0.1 g daily). Following targeted antimicrobial therapy, the patient’s fever resolved, and his right-sided headache, blurred vision, and IOP significantly improved (post-treatment IOP: right, 22 mmHg; left, 20 mmHg). He was discharged on oral doxycycline (100 mg twice daily) and reported complete symptom resolution at a 10-day follow-up.

## DISCUSSION

*C. psittaci* is a strictly intracellular, gram-negative bacterium primarily transmitted to humans through the inhalation of aerosolized droppings or respiratory secretions from infected birds. Human psittacosis typically presents as an atypical pneumonia after a 5-to-14-day incubation period (1), with non-specific symptoms such as high fever, non-productive cough, dyspnea, fatigue, and chills (2, 3). However, *C. psittaci* exhibits broad tissue tropism and can cause severe systemic disease, disseminating hematogenously to the CNS, liver, endocardium, and kidneys. While CNS involvement can lead to meningitis, encephalitis, and cranial nerve palsies, these symptoms usually manifest symmetrically (4). The presentation of strict unilateral headache and unilateral elevated intraocular pressure as the initial and predominant symptoms of psittacosis is highly atypical and expands the known clinical spectrum of this infection (5).

The recommended first-line treatment for psittacosis is tetracycline. In this case, the patient initially received only intraocular pressure-lowering agents for his ocular symptoms. After the tNGS result confirmed *C. psittaci* infection, targeted antimicrobial therapy was initiated. The patient received intravenous omadacycline (0.1 g daily), a novel tetracycline with good lung penetration, followed by oral doxycycline (100 mg twice daily) upon discharge. Clinical improvement in headache, blurred vision, and intraocular pressure was observed after the start of omadacycline. This case illustrates how timely molecular diagnosis can guide appropriate antimicrobial therapy and improve clinical outcomes.

## DIAGNOSTIC APPROACHES FOR *C. PSITTACI*

The diagnosis of *C. psittaci* has historically been challenging due to the fastidious nature of the organism and the non-specific clinical presentation of the disease, frequently leading to underdiagnosis or misdiagnosis. Laboratory diagnostics generally fall into three categories: culture, serology, and molecular testing.

Historically, isolation of *C. psittaci* in cell culture (such as McCoy or HeLa cells) or embryonated chicken eggs was the gold standard. However, culture is highly discouraged for routine diagnostics. *C. psittaci* is highly infectious and poses a severe laboratory-acquired infection risk via aerosolization; therefore, culture must be performed strictly within biosafety level 3 (BSL-3) facilities. Furthermore, the organism is slow-growing, taking several days to weeks, rendering it clinically impractical for guiding acute patient management.

A practical point worth emphasizing is that *C. psittaci* cannot be reliably visualized by conventional Gram stain. As an obligate intracellular bacterium with a cell envelope lacking classical peptidoglycan, *C. psittaci* stains poorly or not at all with Gram stain. Even with specialized stains such as Giemsa or Macchiavello, the sensitivity for detecting chlamydial inclusions directly in clinical specimens (including CSF, BALF, and sputum) is very low. Therefore, negative findings on routine Gram stain of CSF or BALF should not discourage clinicians from considering psittacosis when clinical and epidemiological features suggest this diagnosis.

Serological testing, particularly the microimmunofluorescence (MIF) test, is the most widely available traditional diagnostic method. A confirmed case typically requires a fourfold or greater increase in antibody titer between acute and convalescent serum samples collected 2–6 weeks apart or an IgM titer of ≥1:16. The major limitation of serology is its retrospective nature; it rarely assists in early clinical decision-making. Additionally, significant cross-reactivity exists between *C. psittaci*, *Chlamydia pneumoniae*, and *Chlamydia trachomatis* due to shared lipopolysaccharide antigens, complicating definitive species identification.

In recent years, molecular diagnostics have become the method of choice for both rapid and accurate identification. Real-time PCR targeting the ompA gene or the 16S rRNA gene offers high sensitivity and specificity, bypassing the safety risks of culture and the delayed timeline of serology. Multiplex respiratory PCR panels occasionally include *C. psittaci*, but many standard commercial panels do not, meaning the clinician must have a high index of suspicion to specifically order the test.

In this case, the highly atypical initial presentation (unilateral headache and ocular hypertension) obscured the classical diagnostic pathway for psittacosis, leading to the utilization of tNGS on BALF. The detection of a high specific sequence count (30,062) for *C. psittaci* provided a definitive and rapid diagnosis that prompted an immediate shift in antimicrobial management, demonstrating the critical utility of advanced molecular diagnostics in resolving cryptic clinical presentations.

Given the increasing use of next-generation sequencing in clinical microbiology, it is important to distinguish between two principal approaches. Metagenomic NGS (mNGS) performs untargeted sequencing of all nucleic acids present in a specimen, including human host DNA and microbial DNA from bacteria, viruses, fungi, and parasites. This hypothesis-free approach offers the advantage of detecting unexpected or novel pathogens but at a higher cost and with greater bioinformatics complexity due to the large proportion of human reads. In contrast, tNGS enriches for specific pathogen gene regions prior to sequencing, typically through multiplex PCR amplification or hybridization probe capture. This targeted approach achieves higher sensitivity for organisms included in the panel, reduces sequencing costs, and simplifies data analysis, but it can only detect pathogens for which specific primers or probes are designed.

It is also important to recognize that tNGS is not a single, standardized assay but rather a methodological category with substantial variability across different platforms and laboratories. Key sources of heterogeneity include the enrichment strategy (amplicon-based vs hybridization capture), the composition and size of the target pathogen panel, sequencing platform and depth, bioinformatics pipelines for host subtraction and taxonomic assignment, and the criteria for reporting positive results. Furthermore, clinical availability of tNGS varies geographically. While commercial tNGS panels such as the one used here are increasingly available in China, they may not be accessible in all healthcare settings internationally. Clinicians interpreting tNGS results should be aware of these technical and logistical variables.

Beyond these technical considerations, the interpretation of tNGS results on non-sterile specimens presents additional challenges. It is important to acknowledge that BALF is not a sterile specimen. The respiratory tract normally harbors a diverse microbial community, and detection of microbial nucleic acids by tNGS does not necessarily equate to active infection. Potential pitfalls include colonization, contamination during specimen collection or laboratory processing, and detection of non-viable organisms. Therefore, tNGS results must always be interpreted in the context of the patient’s clinical presentation, inflammatory markers, and imaging findings. In this case, the high clinical suspicion for atypical pneumonia, elevated inflammatory markers, and characteristic chest CT findings all supported the pathogenic role of *C. psittaci*.

One of the challenges of tNGS on non-sterile specimens is determining whether a detected organism is clinically relevant or an incidental finding. In this case, the tNGS report provided both qualitative detection and quantitative pathogen load (copies/mL) using an internal standard spiked into each sample. The tNGS assay used here was performed using the DRseq panel (Yuguo Biotechnology, Beijing, China), which is designed for pathogen identification and resistance gene detection. Sequencing was conducted on the Illumina NextSeq 550 platform, and bioinformatics analysis involved subtraction of human host sequences followed by alignment to a curated microbial reference database. Quantitative analysis was achieved by spiking each BALF sample with a known quantity of internal standard synthetic DNA prior to sequencing. The absolute pathogen load (copies/mL) was calculated based on the ratio of *C. psittaci*-specific reads to internal standard reads. Normalized read counts were expressed as RPM to account for variations in total sequencing depth across samples. The reporting laboratory (Yuguo Biotechnology) has established internal pathogenic thresholds based on analytical validation studies, with a limit of detection of approximately 100–500 copies/mL for most respiratory pathogens. For *C. psittaci*, the specific sequence count (30,062) and pathogen load (6.39 × 10⁴ copies/mL) were orders of magnitude above this threshold, strongly supporting its etiological role. Importantly, there is no universal cutoff for tNGS interpretation across different platforms and laboratories. Current expert consensus recommends integrating quantitative tNGS data with clinical, laboratory, and radiological findings on a case-by-case basis. This case exemplifies that approach.

In conclusion, this case highlights that *C. psittaci* infection can present with highly atypical, extrapulmonary initial symptoms, such as unilateral headache and ocular hypertension. When patients present with unexplained systemic and neurological or ophthalmic symptoms accompanied by pulmonary imaging abnormalities, clinicians must maintain a broad differential diagnosis. Furthermore, this case underscores the indispensable role of modern molecular diagnostics, such as tNGS, in rapidly identifying fastidious pathogens that evade traditional culture and serological methods, thereby preventing delayed or inappropriate treatment.

## SELF-ASSESSMENT QUESTIONS

1. Which of the following is considered the primary reason why routine bacterial culture is not recommended for the diagnosis of *Chlamydia psittaci*?
  - The organism grows too rapidly, easily overgrowing other clinically relevant respiratory flora.
  - The organism is an obligate intracellular bacterium that poses a severe aerosol hazard, requiring BSL-3 facilities.
  - The organism lacks a cell wall, rendering it completely unculturable in all known *in vitro* media.
  - Culture methods cannot differentiate between *C. psittaci* and viral respiratory pathogens.
2. Which of the following is a major limitation of using microimmunofluorescence testing of sera to diagnose atypical pneumonia?
  - It requires a cerebrospinal fluid sample to be accurate.
  - It is overly sensitive, resulting in a high rate of false positives from environmental dust.
  - It often shows cross-reactivity with other *Chlamydia* species (e.g., *C. pneumoniae*) and is retrospective, rarely aiding acute management.
  - It can only detect the pathogen during the first 48 hours of symptom onset.
3. In a patient with an unexplained, atypical respiratory infection accompanied by unusual extrapulmonary symptoms, what is the primary advantage of utilizing tNGS over a standard pathogen-specific PCR?
  - tNGS is significantly cheaper and more widely available in rural clinics than standard PCR.
  - tNGS relies on a hypothesis-free or broadly targeted approach, allowing for the detection of unexpected pathogens without needing specific clinical suspicion to order the correct primer.
  - tNGS automatically determines the antibiotic susceptibility profile of the organism.
  - tNGS is the only method approved to detect viral coinfections alongside bacterial pathogens.

## ANSWERS TO SELF-ASSESSMENT QUESTIONS

1. Which of the following is considered the primary reason why routine bacterial culture is not recommended for the diagnosis of *Chlamydia psittaci*?
  - The organism grows too rapidly, easily overgrowing other clinically relevant respiratory flora.
  - The organism is an obligate intracellular bacterium that poses a severe aerosol hazard, requiring BSL-3 facilities.
  - The organism lacks a cell wall, rendering it completely unculturable in all known *in vitro* media.
  - Culture methods cannot differentiate between *C. psittaci* and viral respiratory pathogens.

Answer: b. *C. psittaci* is highly infectious and transmission occurs easily via aerosolized particles. Due to the high risk of severe laboratory-acquired infections, culturing the pathogen is strictly restricted to BSL-3 facilities and is not utilized for routine clinical diagnostics. Advanced molecular testing has largely replaced culture for this reason.

1. A patient presents with atypical pneumonia. Paired acute and convalescent serology via MIF is ordered. Which of the following is a major limitation of this diagnostic approach?
  - It requires a cerebrospinal fluid sample to be accurate.
  - It is overly sensitive, resulting in a high rate of false positives from environmental dust.
  - It often shows cross-reactivity with other *Chlamydia* species (e.g., *C. pneumoniae*) and is retrospective, rarely aiding acute management.
  - It can only detect the pathogen during the first 48 hours of symptom onset.

Answer: c. While MIF is a traditional serological standard, it requires paired sera taken weeks apart to demonstrate a fourfold titer rise, making it useless for acute clinical decision-making. Furthermore, the shared lipopolysaccharide antigens among the Chlamydiaceae family frequently lead to cross-reactivity, complicating species-level identification.

1. In a patient with an unexplained, atypical respiratory infection accompanied by unusual extrapulmonary symptoms, what is one potential advantage of utilizing tNGS over a standard pathogen-specific PCR?
  - tNGS is significantly cheaper and more widely available in rural clinics than standard PCR.
  - tNGS relies on a hypothesis-free or broadly targeted approach, allowing for the detection of unexpected pathogens without needing specific clinical suspicion to order the correct primer.
  - tNGS automatically determines the antibiotic susceptibility profile of the organism.
  - tNGS is the only method approved to detect viral coinfections alongside bacterial pathogens.

Answer: b. Unlike traditional PCR, which requires the clinician to suspect a specific pathogen to order the correct targeted assay, broad-panel tNGS can detect a wide array of pathogens simultaneously without an a priori hypothesis. However, it is important to note that tNGS has limitations, including higher cost, variable availability, and the need for careful interpretation of results on non-sterile specimens.

**TAKE-HOME POINTS**

- **Atypical presentations obscure diagnosis.** *C. psittaci* can present with highly unusual extrapulmonary initial symptoms—such as unilateral headache and ocular hypertension—which can delay diagnosis if the clinician only looks for primary CNS or ophthalmic etiologies.
- **The power of advanced diagnostics.** When patients present with cryptic systemic symptoms alongside pulmonary infiltrates and standard microbiological tests are negative, tNGS or mNGS serves as a critical, unbiased tool to identify fastidious pathogens.
- **Limitations of traditional assays. **Routine culture of *C. psittaci* is impractical and dangerous due to BSL-3 requirements, while serology is often retrospective and prone to cross-reactivity. Molecular methods are the modern standard for acute diagnosis.
- **Importance of social history.** Even in the era of advanced molecular diagnostics, a thorough patient history regarding environmental exposures (such as recent contact with live poultry or pet birds) remains a cornerstone for suspecting zoonotic infections like psittacosis.

## Data Availability

The data sets for this article are not publicly available due to concerns regarding participant/patient anonymity. Requests to access the data sets should be directed to the corresponding author.
